# Identification of Novel *USH2A* Mutations in a Consanguineous Chinese Family With Usher Syndrome

**DOI:** 10.1155/humu/6391770

**Published:** 2025-02-11

**Authors:** Haolin Wang, Bo Wei, Jiaxin Guo, Xiawei Wu, Tongdan Zou, Ting Wang, Tiantian Zhang, Bo Gong, Jilong Hao, Houbin Zhang, Le Wang

**Affiliations:** ^1^The Key Laboratory for Human Disease Gene Study of Sichuan Province and Center for Medical Genetics, Sichuan Provincial People's Hospital, School of Medicine, University of Electronic Science and Technology of China, Chengdu, Sichuan, China; ^2^Department of Neurosurgery, China-Japan Union Hospital of Jilin University, Changchun, Jilin, China; ^3^Department of Ophthalmology, The First Hospital of Jilin University, Changchun, China; ^4^Research Unit for Blindness Prevention of Chinese Academy of Medical Sciences (2019RU026), Sichuan Academy of Medical Sciences and Sichuan People's Hospital, Chengdu, Sichuan, China

## Abstract

Usher syndrome (USH) is a rare genetic disease characterized by sensorineural deafness and blindness called retinitis pigmentosa, and it is inherited in an autosomal recessive pattern with a prevalence of four to 17 per 100,000 people worldwide. In this study, a consanguineous Chinese family with USH, including two affected individuals and five unaffected individuals, was recruited. All subjects received an ophthalmic examination and an auditory examination. The two USH patients exhibited severe early-onset hearing and vision loss. DNA samples from the two USH patients were analyzed using whole-exome sequencing. A novel homozygous frameshift mutation (NM_206933.4:c.6379_6380delinsC, p.G2127Pfs∗25) in *USH2A*, resulting in a truncated *USH2A* protein lacking 3051 amino acids, was identified in the proband. In addition, novel compound mutations in *USH2A* (one allele harboring NM_206933.4:c.9958G>T, p.G3320C; NM_206933.4:c.8284C>G, p.P2762A; and the other NM_206933.4:c.6379_6380delinsC; p.G2127Pfs∗25) were identified in the other affected individual. In silico analysis predicts that while the p.G3320C mutation has little impact on the local structure around the mutation site, the p.P2762A substitution may alter the protein's interaction with its binding partners. In addition, p.G2127Pfs∗25 causes a truncation of a major portion of the protein that severely disrupts the protein structure and results in the loss of its function. In conclusion, this study identified novel USH mutations in *USH2A* and expanded the spectrum of disease-associated variants in the *USH2A* gene, which will promote the molecular screening of genetic mutations in USH patients.

## 1. Introduction

Usher syndrome (USH) is a rare genetic disease characterized by deafness and vision loss. In USH patients, while deafness results from sensorineural inner ear dysfunction, vision loss is caused by retinitis pigmentosa (RP) [1]. USH is inherited in an autosomal recessive pattern with a prevalence of four to 17 per 100,000 people worldwide [2, 3]. USH is a highly heterogeneous disease. Based on the severity of the hearing loss, the status of vestibular dysfunction, and the onset age of RP, USH has been classified into three major clinical subtypes (from severe to less severe): Type 1 (USH1), Type 2 (USH2), and Type 3 (USH3) [4]. Type 1 and Type 2 are the most common forms of USH, accounting for 95% of cases [5]. Until 2015, 16 USH-associated genes/loci have been identified [6]. Nine of these 16 genes/loci are involved in *USH1*, three in *USH2*, and two in *USH3*. The remaining two are not specified. Subsequently, *USH1J* was excluded from the list of USH-associated genes [7]. More recently, an additional nine genes/loci have been related to USH; however, further evidence is required for verification, including *USH1M*, *USH3*, *CEP78*, *CEP250*, *ABDH12*, *ARSG*, and three loci (*USH1E*, *USH1H*, and *USH1K*) [8]. To date, nine genes have been confirmed to cause USH, with *MYO7A*, *USH1C*, *CDH23*, *PCDH15*, and *SANS* associated with USH1; *USH2A*, *ADGRV1*, and *WHRN* with USH2; and *CLRN1* with USH3 [8]. These genes are essential for the normal functioning of hair cells and photoreceptors [6]. Mutations in any of these genes could cause cochlear hair cell dysfunction and photoreceptor degeneration. No effective treatment is currently available to rescue auditory or visual dysfunction in USH patients. Gene therapy and gene editing, however, appear promising [9]. Due to this lack of effective treatment, mutation screening is crucial for avoiding sick newborns.

Mutations in *USH2A* are the most common cause of USH, accounting for 75%–90% of USH2 cases [10–12]. *USH2A* has 72 exons and encodes a large transmembrane protein containing 5202 amino acid residues with a calculated molecular weight of ~580 kDa. The USH2A protein is specifically expressed in the photoreceptor periciliary membrane complex and the developing hair cell ankle link complex [13–16]. To date, 2567 distinct public DNA variants in *USH2A* have been reported (https://databases.lovd.nl/shared/genes/USH2A), and more than 600 mutations in *USH2A* have been associated with USH2 [17–19]. Additionally, *USH2A* may cause nonsyndromic autosomal recessive retinitis pigmentosa (nsRP), accounting for 12%–25% of all RP cases in the United States [20, 21].

In this study, we report on a consanguineous Chinese family with USH consisting of two patients. Whole-exome sequencing (WES) was used to identify pathogenic mutations. One novel homozygous mutation in *USH2A* was identified in the proband, and additional novel compound heterozygous mutations in *USH2A* were identified in the other affected individuals in the family.

## 2. Methods and Materials

### 2.1. Subjects

A Chinese family with USH, including two affected individuals and five unaffected individuals, was recruited from the First Hospital of Jilin University in Changchun, China. The study was conducted in conformity with the tenets of the Declaration of Helsinki and approved by the Institutional Review Boards of Sichuan Provincial People's Hospital, China (approval number: 2020-7), and the First Hospital of Jilin University, China (approval number: 23K289-001). Written informed consent was obtained from all participants before inclusion.

### 2.2. Clinical Diagnosis

All participants, including one proband and six other family members, underwent comprehensive ophthalmic examinations and auditory examinations at the First Hospital of Jilin University (Changchun, China), including routine eye tests, spectral-domain optical coherence tomography (OCT), electroretinography (ERG), audiological examination, and acoustic immittance examination. One family member (VI-2), the proband, was diagnosed with USH2. The criteria for USH2 diagnosis include RP (beginning with night blindness, progressive loss of peripheral vision, attenuated blood vessels, and tunnel vision or complete vision loss in the late stage) and congenital bilateral sensorineural hearing loss (relatively mild at low frequencies and severe at high frequencies). Another family member (VI-3) was diagnosed with autosomal recessive retinitis pigmentosa (ARRP) and early stage of hearing impairment (very mild hearing impairment at high frequency in 1 year). All other family members tested exhibited no vision or hearing loss. The clinical diagnosis information is summarized in Table S1.

### 2.3. DNA Isolation

Peripheral blood samples were collected in EDTA tubes from all seven members of this family. Genomic DNA was extracted using a blood DNA extraction kit according to the manufacturer's instructions (Tiangen Biotech, China). The DNA samples were stored at −80°C until use.

### 2.4. WES

The DNA from individuals VI-2 (the proband) and VI-3 was analyzed using WES with a mean read depth of 100x. The samples were prepared following the Illumina standard procedure (Illumina Inc.), as previously described [22]. The sequencing was performed on an Illumina HiSeq4000 platform (Illumina Inc.), and paired-end 150 bp reads were obtained.

### 2.5. Mutation Identification and Data Analysis

The variants in *USH2A* were identified using the following databases: dbSNP138 (https://www.ncbi.nlm.nih.gov/snp/), the 1000 Genomes Project (https://www.internationalgenome.org/), the Exome Aggregation Consortium (https://gnomad.broadinstitute.org/), HGMD (http://www.hgmd.cf.ac.uk/ac/index.php), other East Asian population databases (https://blog.nus.edu.sg/sshsphphg/singapore-genome-variation/), ClinVar (https://www.ncbi.nlm.nih.gov/clinvar/), and a USH database belonging to the retinal and hearing impairment genetic variant databases (https://databases.lovd.nl/shared/genes/USH2A). To confirm the candidate mutations, polymerase chain reaction (PCR) was used to amplify DNA fragments encompassing mutations from seven available family members, followed by sequencing on a 3730 ABI DNA sequencer (Thermo Fisher Scientific, United States). In addition, the candidate mutations were compared against our in-house database comprising the exome sequencing results of 400 normal healthy controls. Online bioinformatics tools, including MutationTaster (https://www.mutationtaster.org/), CADD (https://cadd.gs.washington.edu/), PROVEAN (http://provean.jcvi.org/index.php), PolyPhen-2 (http://genetics.bwh.harvard.edu/pph2/), Panther (http://www.pantherdb.org/tools/), and Sorting Intolerant from Tolerant (SIFT) (https://sift.bii.a-star.edu.sg/), were used to predict the potential pathogenic effects of variants identified in *USH2A*. The procedure for the identification of the mutations is shown by a flowchart in Figure S1. The mutation's information has been deposited in ClinVar (accession number: SCV005380295).

The 3D structure of the target protein was constructed using homology modeling on the SWISS-MODEL web server (https://swissmodel.expasy.org/). The amino acid sequence (UniProt ID: O75445) was provided in FASTA format, allowing SWISS-MODEL to automatically select A0A7L3CFB1.1.A USH2A as the best matching template, based on sequence similarity and template quality, with an identity of 70.81%. The final model was generated by aligning the target sequence to the selected template, followed by a refinement procedure within the SWISS-MODEL platform. The complete structural model of the truncation mutant protein was generated with AlphaFold 3 (https://alphafoldserver.com/). The c.6379_6380delinsC; p.G2127Pfs∗25 mutant USH2A protein containing 2150 amino acids was submitted to the AlphaFold 3 online server for structure prediction. The best matching template was automatically selected, and four models were produced. The model with the highest score (0.55) was selected for display.

## 3. Results

### 3.1. Clinical Phenotype

**Figure 1:**
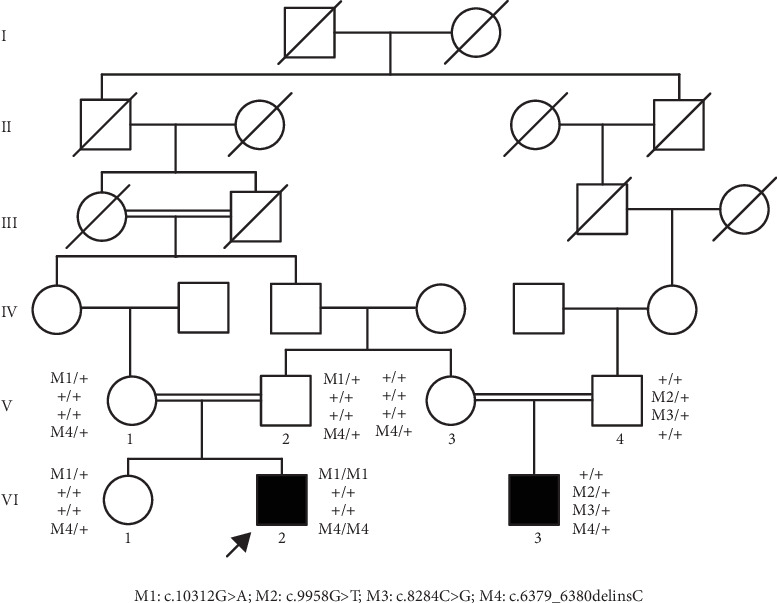
Pedigree of the Chinese family with USH. Circles and squares represent females and males, respectively. Open and filled symbols indicate unaffected and affected individuals, respectively. Crossed symbols indicate deceased family members. The arrow indicates the proband. M1, M2, M3, and M4 represent the mutations of c.10312G>A, c.9958G>T, c.8284C>G, and c.6379_6380delinsC in *USH2A* identified in this family, respectively.

**Figure 2:**
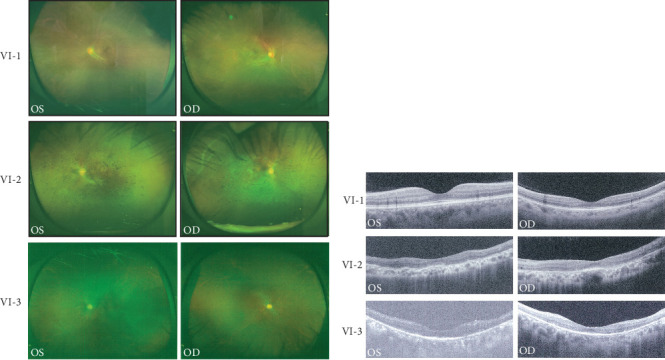
Fundus photographs and optical coherence tomography (OCT) images. (a) Fundus images of the proband (VI-2), the proband's sister (VI-1), and individual VI-3. (b) OCT images of the proband (VI-2), the proband's sister (VI-1), and individual VI-3. OS, left eye; OD, right eye.

**Figure 3:**
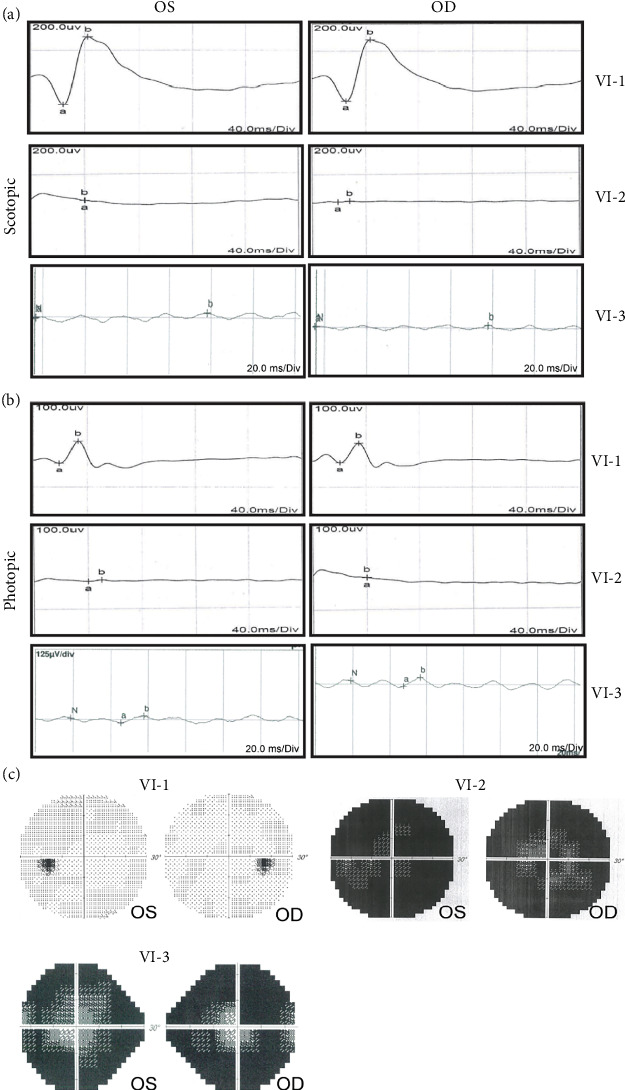
Electroretinography recordings and visual field test of the proband (VI-2), the proband's sister (VI-1), and individual IV-3. (a) Full-field scotopic, (b) photopic, and (c) visual field test. OS, left eye; OD, right eye.

**Figure 4:**
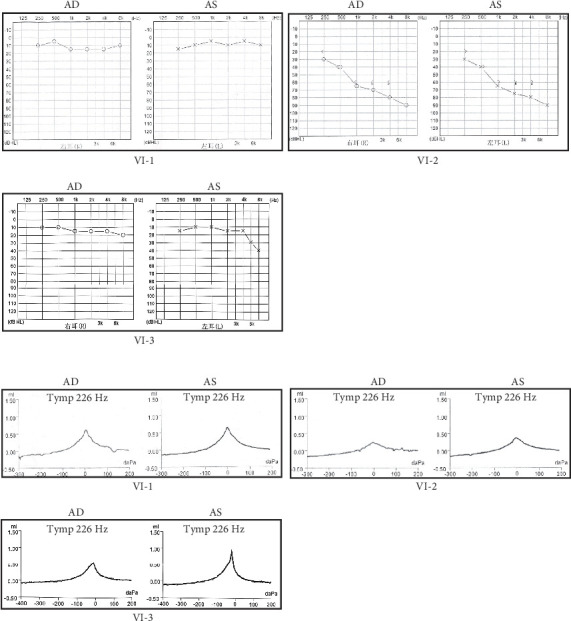
Audiologic and acoustic immittance tests. (a) Pure-tone audiometry and (b) acoustic immittance examination for the proband (VI-2) and two other family members (VI-1 and VI-3). AS, left ear; AD, right ear.

A Chinese family with USH, including seven members with a history of consanguineous marriage, was enrolled in this study. The pedigree diagram is presented in Figure 1. Two family members were affected. The analysis of the pedigree suggests a pattern of autosomal recessive inheritance in this family. The proband began to experience night blindness at the age of eight and was diagnosed with USH at the age of 15. Individual VI-3 complained about night blindness at the age of 15 and was diagnosed with USH at the age of 34. Upon recruitment to this study, fundus examination revealed severe RP symptoms in the proband (Aged 36) and individual VI-3 (Aged 34), including attenuated retinal blood vessels and pale optic disks, whereas the proband's sister (VI-1) exhibited normal fundus (Figure 2(a)). OCT showed a thinner and disordered outer nuclear layer in the proband (VI-2) and individual VI-3 (Figure 2(b) and Table S2). The ERGs of the proband (VI-2) and individual VI-3 under scotopic and photopic conditions were barely recordable, indicating severe rod-cone dysfunction (Figures 3(a) and 3(b)). Consistently, the visual field tests indicated only residual vision in the central region of the proband and individual VI-3 (Figure 3(c)). By contrast, the vision of unaffected individuals and other unaffected members was normal. The pure-tone audiogram revealed that unaffected family members, such as individual VI-1, had normal hearing thresholds, whereas the proband had elevated hearing thresholds in the test range (250–8k Hz), particularly in the high-frequency range, indicating a sensorineural hearing loss in the proband (Figure 4(a)). The pure-tone audiogram also showed that individual VI-3 had normal hearing in the left ear and nearly normal hearing in the right ear which was slightly affected at high frequencies. An acoustic immittance test showed that the unaffected family member (VI-1) had a normal compliance value in both ears. By contrast, the affected individual (VI-2) had an abnormal compliance value (0.22 mL) in the left ear, with a compliance value at the low end of the normal range (0.36 mL) in the right ear (Figure 4(b)), indicating impaired middle ear function. Individual VI-3 performed well in the acoustic immittance test, indicating normal middle ear function.

### 3.2. WES and Data Analysis

To identify the genetic mutations associated with USH in this family, DNA from affected individuals (VI-2 and VI-3) was analyzed using WES. A total of 72,339 single-nucleotide polymorphisms (SNPs) and indels were obtained. In the proband (VI-2), 27,192 variants in the coding regions, untranslated regions (UTRs) (5⁣′UTR and 3⁣′UTR), and splice junctions were obtained, including 13,641 nonsynonymous SNPs, 12,977 synonymous SNPs, and 574 indels. Meanwhile, in the other affected individual (VI-3), 27,424 genetic variants were obtained in the coding regions, UTRs (5⁣′UTR and 3⁣′UTR), and splice junctions, including 13,843 nonsynonymous SNPs, 12,977 synonymous SNPs, and 604 indels. To identify the disease-associated gene in this family, common variants with high frequencies in HGMD, ClinVar, dbSNP138, the 1000 Genomes Project, the Exome Aggregation Consortium, and other East Asian population databases were filtered out. Subsequently, variants located in introns and UTRs, synonymous SNPs, and nonframeshift indels were also filtered out, as they usually do not alter gene function. The remaining variants (shown in Tables S3 and S4) were compared with the variants reported in USH-related databases. Because the proband's parents and siblings presented with normal vision and hearing, a recessive genetic model was applied to analyze the remaining variants. To define the pathogenic variants, the homozygous and compound heterozygous variants were closely followed. As a result, two homozygous mutations in *USH2A*, including one missense mutation (NM_206933.4:c.10312G>A (p.A3438T)) and one frameshift mutation (NM_206933.4:c.6379_6380delinsC (p.G2127Pfs∗25)), were identified in the proband (VI-2). Three additional heterozygous mutations in individual VI-3 were identified, including two missense mutations (NM_206933.4:c.9958G>T (p.G3320C) and NM_206933.4:c.8284C>G (p.P2762A)) and one frameshift mutation (NM_206933.4:c.6379_6380delinsC (p.G2127Pfs∗25)). The novel homozygous frameshift mutation (c.6379_6380delinsC (p.G2127Pfs∗25)) results in the truncation of *USH2A*, which likely causes the loss of function. Thus, *USH2A* was considered the candidate pathogenic gene for USH symptoms in this family.

### 3.3. Verification of Mutations in the *USH2A* Gene

**Figure 5:**
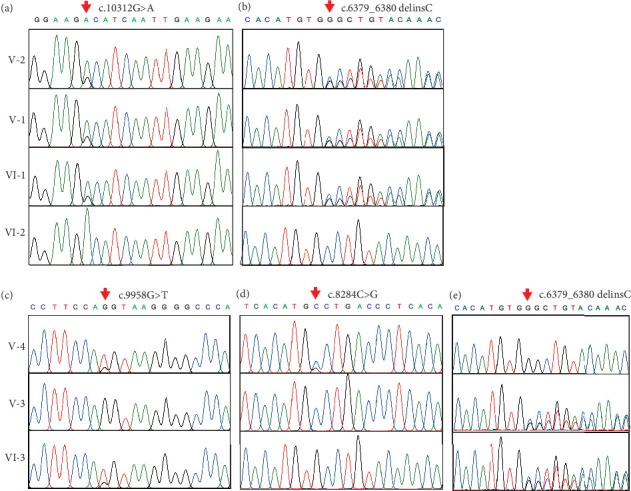
Verification of the mutations in *USH2A* using Sanger sequencing. Partial sequences of *USH2A* Exons (a) 52 and (b) 33 in the proband (VI-2), individual VI-1, and their parents (V-1 and V-2). Partial sequence of *USH2A* Exons (c) 50, (d) 42, and (e) 33 in individual VI-3 and his parents (V-3 and V-4). Red arrows indicate the mutation sites, including c.10312G>A, c.6379_6380delinsC, c.9958G>T, and c.8284C>G.

Sanger sequencing was used to verify these three mutations for all seven available family members. The results confirmed that the proband harbored homozygous mutations in both c.10312G>A (p.A3438T) and c.6379_6380delinsC (p.G2127Pfs∗25 in *USH2A* (Figures 5(a) and 5(b)). The proband's parents (V-1 and V-2) and his sister (VI-1) were heterozygous carriers of these two mutations. The mutation c.10312G>A (p.A3438T) was reported previously in a family with biliary atresia [23] and ciliary defects but without symptoms in the eye or ear, which suggests that this single missense mutation (c.10312G>A (p.A3438T)) was not the mutation associated with USH. Rather, the frameshift mutation c.6379_6380delinsC (p.G2127Pfs∗25) was responsible for USH in the proband. Sanger sequencing also confirmed that individual VI-3 carried three heterozygous mutations in *USH2A*, including c.6379_6380delinsC (p.G2127Pfs∗25), c.9958G>T (p.G3320C), and c.8284C>G (p.P2762A) (Figures 5(c), 5(d), and 5(e)). In addition, individual VI-3's mother (V-3) carried heterozygous c.6379_6380delinsC (p.G2127Pfs∗25) (Figure 5(e)), and his father (V-4) carried two heterozygous mutations: c.9958G>T (p.G3320C) and c.8284C>G (p.P2762A) (Figures 5(c) and 5(d)). Thus, individual VI-3 carried compound mutations in *USH2A*, with one allele harboring c.6379_6380delinsC (p.G2127Pfs∗25), which was inherited from his mother, and the other allele, c.9958G>T (p.G3320C) and c.8284C>G (p.P2762A), which was inherited from his father. As c.9958G>T (p.G3320C) and c.8284C>G (p.P2762A) have been linked to USH [24], and c.6379_6380delinsC (p.G2127Pfs∗25) causes the truncation of approximately one-third of the USH2A protein, the compound variants in individual VI-3 result in loss of function for both *USH2A* alleles. c.9958G>T (p.G3320C), c.8284C>G (p.P2762A), or c.6379_6380delinsC (p.G2127Pfs∗25) were not identified in any of 400 unrelated healthy control individuals. Therefore, the disease phenotype in the proband results from the homozygous novel mutation (c.6379_6380delinsC (p.G2127Pfs∗25)), whereas the USH symptoms in individual VI-3 can be attributed to the compound heterozygous *USH2A* mutations (c.9958G>T (p.G3320C); c.8284C>G (p.P2762A)/c.6379_6380delinsC (p.G2127Pfs∗25)).

### 3.4. In Silico Sequence and Structural Analysis

**Figure 6:**
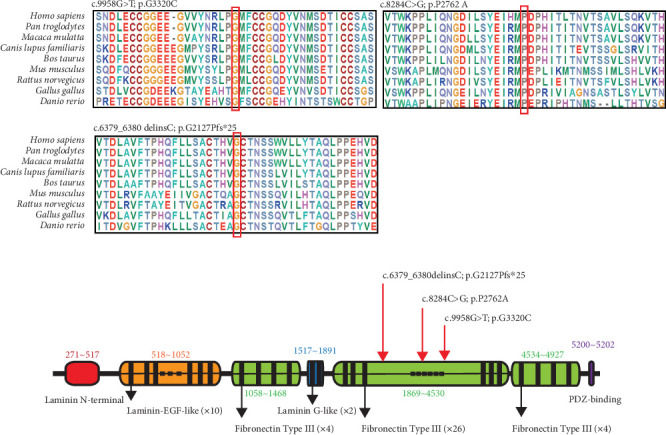
In silico analysis of the *USH2A* protein. (a) Multispecies alignment for *USH2A*. (b) Domain structure of human *USH2A*. The positions of the mutations identified in this study are indicated by red arrows.

**Figure 7:**
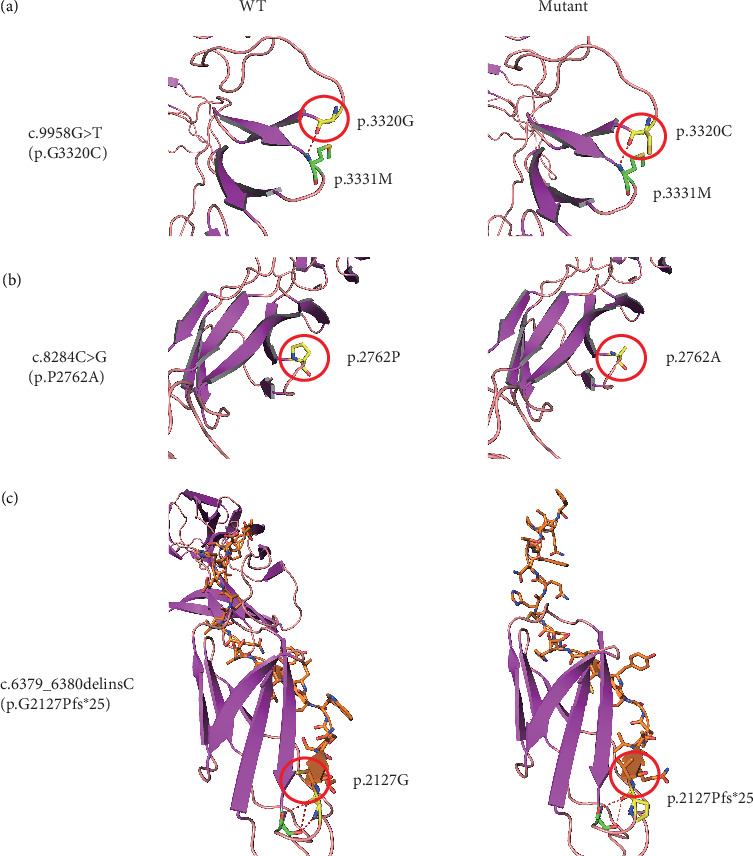
Mutant *USH2A* modeled using SWISS-MODEL. (a–c) Partial wild-type and mutant *USH2A* protein structures modeled using SWISS-MODEL. Mutant amino acid residues for variants (a) c.9958G>T, (b) c.8284C>G, and (c) c.6379_6380delinsC and their corresponding original amino acid residues marked by red circles. Hydrogen bonds are indicated by dotted red lines.

To gain insight into the functional significance of the *USH2A* genetic mutations identified in this family, a multispecies sequence alignment was performed (Figure 6(a)). The results show that c.9958G, c.8284C, and c.6379_6380 are highly conservative among different species, suggesting their critical function in *USH2A*. The USH2A protein consists of multiple conserved domains, including one laminin N-terminal domain, 10 laminin EGF-like domains, two laminin G-like domains, 34 Fibronectin Type III domains, and one PDZ-binding domain (Figure 6(b)). While the mutations c.6379_6380delinsC and c.8284C>G are located within the seventh and 14th Fibronectin Type III domains, respectively, the mutation c.9958G>T is located in the interval between the 18th and 19th Fibronectin Type III domains. The protein structures modeled using SWISS-MODEL showed that one hydrogen bond was predicted to be located between p.G3320 and p.M3331, which is not affected by the substitution of p.G3320 with C (Figure 7(a)). Single amino acid substitution p.P2762A (Figure 7(b)) does not change its formation of hydrogen bonds with adjacent amino acids. Rather, the substitution alters the position of the sequence following this mutation. Figure 7(c) shows that three hydrogen bonds were predicted to form between p.G2127 and p.T2124, but the frameshift mutation c.6379_6380delinsC disrupted one of the three hydrogen bonds, which may have destabilized the protein. Moreover, the mutant protein prematurely terminated 24 amino acid residues after p.P2127 to produce a truncated protein. The structure of the whole truncated mutant protein predicted by AlphaFold is illustrated in Figure S2.

## 4. Discussion

In this study, *USH2A* mutations were identified in a consanguineous Chinese family with USH. *USH2A* is a large transmembrane protein required for the formation of the hair bundle ankle in the inner ear. *USH2A* plays a critical role in the development of cochlear hair cells [25]. In photoreceptors, *USH2A* is a structural protein involved in maintaining the membrane complex by regulating intracellular protein transport between photoreceptor outer segments and inner segments [25].

More than 600 genetic mutations in *USH2A* have been reported to be associated with USH, accounting for over 70% of USH2 cases across multiple populations worldwide [26]. This study identified three USH-associated mutations, including one novel frameshift mutation (c.6379_6380delinsC) and two reported missense mutations (c.9958G>T and c.8284C>G). Previously, c.9958G>T and c.8284C>G have been identified in a family with ARRP. Both mutations were homozygous in one affected individual [24]. It remains unclear which variant predominantly causes the disease phenotype. Both are likely required for the pathogenesis of USH, as neither of them has been identified in USH patients alone. p.P2762A or p.G3320C might slightly alter the protein's function but is not sufficient to damage the cell function or affect the cell's viability. The SWISS-MODEL predicts that p.G3320C does not affect the hydrogen bond between this amino acid and its adjacent p.M3331. However, p.P2762A is predicted to shift the peptide sequence following this amino acid residue (Figure 7(b)). Both p.P2762A and p.G3320C mutations are located in the large domain containing the stretches of 26 Fibronectin Type III domains. They may synergistically alter the domain structure, thereby compromising the function of the protein. A prior study showed that patients carrying two truncation mutations in *USH2A* have a younger onset age and faster progression of hearing impairment and vision loss compared to those with one or two alleles encoding missense mutations [19]. The patient carrying the homozygous double missense mutation (p.P2762A and p.G3320C) only had RP rather than syndromic USH [24], also implying that the double missense mutation has a milder impact on the protein function than the truncation mutation. Thus, in this study, individual VI-3 carrying the double-missense mutation allele had an older onset age for impaired vision than the proband with a homozygous truncation mutation (aged 15 vs. 8 years old). In addition, the hearing of VI-3 was slightly affected at the time of the test (Aged 30–40 years). Thus, the *USH2A* gene harboring these missense mutations may retain some residual function.

The novel frameshift mutation (c.6379_6380delinsC (p.G2127Pfs∗25)) causes the premature termination of translation, resulting in a shorter protein isoform with the deletion of 27 Fibronectin Type III domains and the PDZ-binding domain. Because approximately two-thirds of the protein is absent in the mutant, the truncated protein could completely lose its function. Consequently, the proband carrying homozygous c.6379_6380delinsC (p.G2127Pfs∗25) had a younger disease onset age compared to his affected cousin (VI-3), who carries compound mutations with one allele harboring the double-missense mutations (c.8284C>G (p.P2762A) and c.9958G>T (p.G3320C)). However, how the truncation mutation and the double missense mutations exactly impact the protein function needs to be investigated by further experiments.

## 5. Conclusions

In conclusion, this study, which focused on a consanguineous Chinese family with USH syndrome, identified three disease-associated mutations in the *USH2A* gene, including two known missense mutations and one novel frameshift mutation. Our findings expand the spectrum of *USH2A* mutations, which will facilitate the molecular diagnosis of USH.

## Data Availability

The data of this study are available from the corresponding authors upon reasonable request.

## Supporting Information

Supporting Information Additional supporting information can be found online in the Supporting Information section. Figure S1: A flowchart showing the mutation identification procedure for this family. Figure S2: The structure of the p.G2127Pfs∗25 mutant protein predicted by AlphaFold 3. Table S1: Clinical information of family members available for test. Table S2: Average thickness of different retinal sections measured by OCT for family members VI-1, VI-2, and VI-3. Table S3: Identified genetic variants of USH genes in the proband (VI-2) after filtering out common variants, variants in introns, and synonymous mutations. Table S4: Identified genetic variants of USH genes in family member VI-3 after filtering out common variants, variants in introns, and synonymous mutations.

## References

[1] Petit C. (2001). USHERSYNDROME: from genetics to pathogenesis. *Annual Review of Genomics and Human Genetics*.

[2] Delmaghani S., El-Amraoui A. (2022). The genetic and phenotypic landscapes of Usher syndrome: from disease mechanisms to a new classification. *Human Genetics*.

[3] Toms M., Pagarkar W., Moosajee M. (2020). Usher syndrome: clinical features, molecular genetics and advancing therapeutics. *Therapeutic Advances in Ophthalmology*.

[4] Davenport S. L., Omenn G. S. The heterogeneity of Usher's syndrome.

[5] Saihan Z., Webster A. R., Luxon L., Bitner-Glindzicz M. (2009). Update on Usher syndrome. *Current Opinion in Neurology*.

[6] Mathur P., Yang J. (2015). Usher syndrome: hearing loss, retinal degeneration and associated abnormalities. *Biochimica et Biophysica Acta*.

[7] Booth K. T., Kahrizi K., Babanejad M. (2018). Variants in CIB2 cause DFNB48 and not USH1J. *Clinical Genetics*.

[8] Castiglione A., Möller C. (2022). Usher syndrome. *Audiology Research*.

[9] Géléoc G. G. S., El-Amraoui A. (2020). Disease mechanisms and gene therapy for Usher syndrome. *Hearing Research*.

[10] Dreyer B., Brox V., Tranebjærg L. (2008). Spectrum of USH2A mutations in Scandinavian patients with Usher syndrome type II. *Human Mutation*.

[11] Hope C. I., Bundey S., Proops D., Fielder A. R. (1997). Usher syndrome in the city of Birmingham--prevalence and clinical classification. *The British Journal of Ophthalmology*.

[12] Thomas Rosenberg M. H., Hauch A.-M., Parving A. (1997). The prevalence of Usher syndrome and other retinal dystrophy-hearing impairment associations. *Clinical Genetics*.

[13] Kremer H., van Wijk E., Märker T., Wolfrum U., Roepman R. (2006). Usher syndrome: molecular links of pathogenesis, proteins and pathways. *Human Molecular Genetics*.

[14] Reiners J., Nagel-Wolfrum K., Jürgens K., Märker T., Wolfrum U. (2006). Molecular basis of human Usher syndrome: deciphering the meshes of the Usher protein network provides insights into the pathomechanisms of the Usher disease. *Experimental Eye Research*.

[15] Michalski N., Michel V., Bahloul A. (2007). Molecular characterization of the ankle-link complex in cochlear hair cells and its role in the hair bundle functioning. *Journal of Neuroscience*.

[16] Grati M. H., Shin J. B., Weston M. D. (2012). Localization of PDZD7 to the stereocilia ankle-link associates this scaffolding protein with the Usher syndrome protein network. *The Journal of Neuroscience*.

[17] Li W., Jiang X.-S., Han D.-M. (2022). Genetic characteristics and variation spectrum of USH2A-related retinitis pigmentosa and usher syndrome. *Frontiers in Genetics*.

[18] Young S. L., Stanton C. M., Livesey B. J., Marsh J. A., Cackett P. D. (2022). Novel biallelic USH2A variants in a patient with usher syndrome type IIA- a case report. *BMC Ophthalmology*.

[19] Zhu T., Chen D. F., Wang L. (2021). USH2A variants in Chinese patients with Usher syndrome type II and non-syndromic retinitis pigmentosa. *The British Journal of Ophthalmology*.

[20] Hartong D. T., Berson E. L., Dryja T. P. (2006). Retinitis pigmentosa. *The Lancet*.

[21] McGee T. L., Seyedahmadi B. J., Sweeney M. O., Dryja T. P., Berson E. L. (2010). Novel mutations in the long isoform of the USH2A gene in patients with Usher syndrome type II or non-syndromic retinitis pigmentosa. *Journal of Medical Genetics*.

[22] Zou T., Wang T., Zhen F., Dong S., Gong B., Zhang H. (2022). Identification of a novel compound heterozygous CYP4V2 variant in a patient with autosomal recessive retinitis pigmentosa. *Biomedical Reports*.

[23] Lam W. Y., Tang C. S., So M. T. (2021). Identification of a wide spectrum of ciliary gene mutations in nonsyndromic biliary atresia patients implicates ciliary dysfunction as a novel disease mechanism. *eBioMedicine*.

[24] Chen X., Sheng X., Liu X. (2014). Targeted next-generation sequencing reveals novel USH2A mutations associated with diverse disease phenotypes: implications for clinical and molecular diagnosis. *PLoS One*.

[25] Liu X., Bulgakov O. V., Darrow K. N. (2007). Usherin is required for maintenance of retinal photoreceptors and normal development of cochlear hair cells. *Proceedings of the National Academy of Sciences of the United States of America*.

[26] Bonnet C., Grati M., Marlin S. (2011). Complete exon sequencing of all known Usher syndrome genes greatly improves molecular diagnosis. *Orphanet Journal of Rare Diseases*.

