# Supplementary material for: Identification of Novel USH2A Mutations in a Consanguineous Chinese Family With Usher Syndrome
Source: Hum Mutat. 2025 Feb 11;2025:6391770. doi: 10.1155/humu/6391770 (PMC12267961; doi:10.1155/humu/6391770)
Supplement: Supporting Information — Additional supporting information can be found online in the Supporting Information section. Figure S1: A flowchart showing the mutation identification procedure for this family. Figure S2: The structure of the p.G2127Pfs∗25 mutant protein predicted by AlphaFold 3. Table S1: Clinical information of family members available for test. Table S2: Average thickness of different retinal sections measured by OCT for family members VI-1, VI-2, and VI-3. Table S3: Identified genetic variants of USH genes in the proband (VI-2) after filtering out common variants, variants in introns, and synonymous mutations. Table S4: Identified genetic variants of USH genes in family member VI-3 after filtering out common variants, variants in introns, and synonymous mutations. [file 6391770.f1.docx]

**Supplementary Materials**

**Supplementary Figure S1**

**
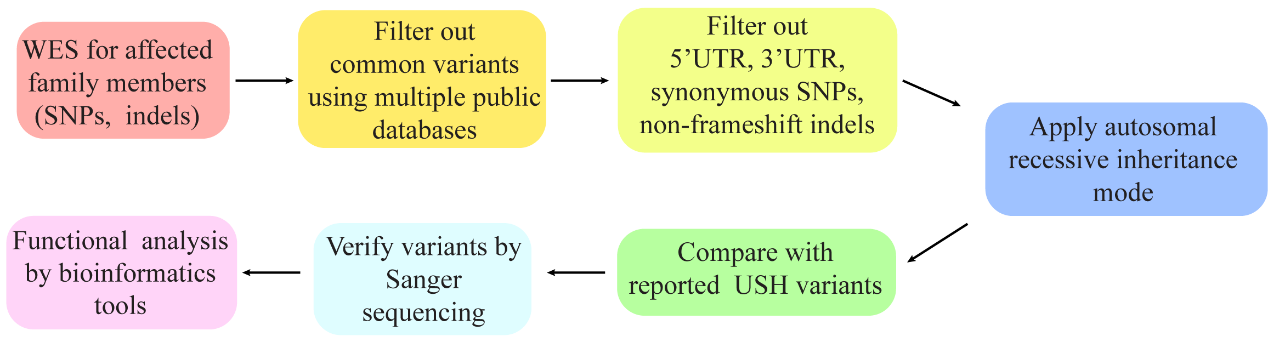
**

**Supplementary Figure S1. Flowchart of pathogenic mutations identification procedure for this family.**

**Supplementary Figure S2**

**
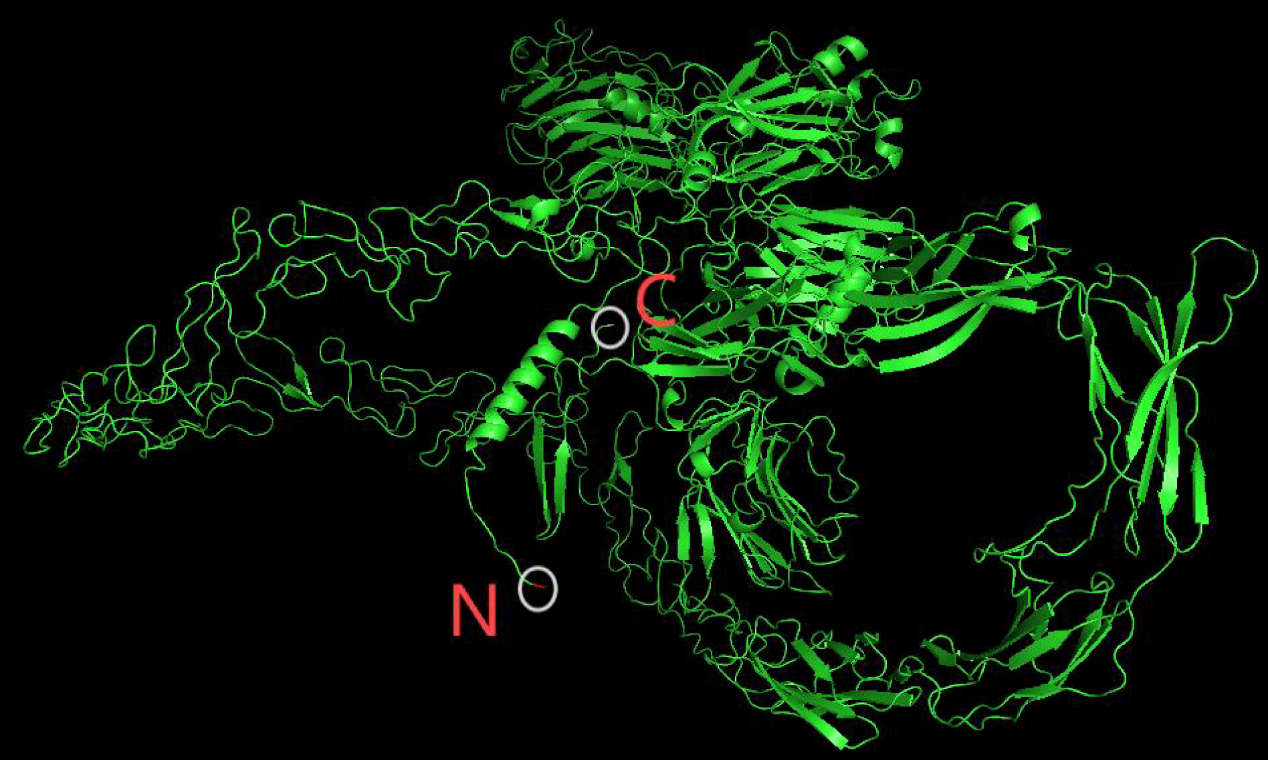
**

**Supplementary Figure S2. Predicted structure of the p.G2127Pfs*25 mutant protein with the truncation of the C-terminus.** N: N-terminus; C: C-terminus.

**Supplementary Table S1. Clinical information of family members.**

| Family  member | Onset age (year) | BCVA | | Fundus appearance | | | | | | ERG | | VF | | Hearing  impairment | | Vestibular  function | |
| --- | --- | --- | --- | --- | --- | --- | --- | --- | --- | --- | --- | --- | --- | --- | --- | --- | --- |
|  |  | OD | OS | OD | | | OS | | | OD | OS | OD | OS | AD | AS | AD | AS |
|  |  |  |  | ONH | AA | PD | ONH | AA | PD |  |  |  |  |  |  |  |  |
| V-1 | NA | 0.6 | 0.4+ | Normal | No | No | Normal | No | No | Normal | Normal | Normal | Normal | Normal | Normal | Normal | Normal |
| V2 | NA | 1.0 | 1.0- | Normal | No | No | Normal | No | No | Normal | Normal | Normal | Normal | Normal | Normal | Normal | Normal |
| V-3 | NA | 1.0 | 1.0 | Normal | No | No | Normal | No | No | Normal | Normal | Normal | Normal | Normal | Normal | Normal | Normal |
| V-4 | NA | 1.0 | 1.0 | Normal | No | No | Normal | No | No | Normal | Normal | Normal | Normal | Normal | Normal | Normal | Normal |
| VI-1 | NA | 1.0 | 1.0 | Normal | No | No | Normal | No | No | Normal | Normal | Normal | Normal | Normal | Normal | Normal | Normal |
| VI-2 | 8 | 0.5 | HM/ 30 cm | Waxy | Yes | Yes | Waxy | Yes | Yes | Significantly  reduced | Significantly  reduced | Tunnel | Tunnel | Mild | Mild | Normal | Normal |
| VI-3 | 15 | 0.5 | 0.2 | Waxy | Yes | Yes | Waxy | Yes | Yes | Significantly  reduced | Significantly  reduced | Tunnel | Tunnel | Normal | Very Mild | Normal | Normal |

Abbreviations: BCVA: best corrected visual acuity; HM: hand motion; OD: right eye; OS: left eye; ONH: optic never head; AA: artery attenuation; PD: pigment deposit; ERG: electroretinography; VF: visual field; AD; right ear; AS: left ear.

**Supplementary Table S2. Measurement of the average thickness in different retinal sections by OCT for family members VI-1, VI-2, and VI-3.**

| Family member | VI-1 | | | | VI-2 | | | | VI-3 | | | |
| --- | --- | --- | --- | --- | --- | --- | --- | --- | --- | --- | --- | --- |
| Eye | OS | | OD | | OS | | OD | | OS | | OD | |
| **Section** | Thick (μm) | Vol (mm^3^) | Thick (μm) | Vol (mm^3^) | Thick (μm) | Vol (mm^3^) | Thick (μm) | Vol (mm^3^) | Thick (μm) | Vol (mm^3^) | Thick (μm) | Vol (mm^3^) |
| Fovea | 230 | 0.181 | 233 | 0.183 | 220 | 0.173 | 205 | 0.161 | 241 | 0.189 | 182 | 0.143 |
| ParaFovea | 310 | 1.95 | 300 | 1.885 | 268 | 1.683 | 257 | 1.618 | 252 | 1.582 | 239 | 1.503 |
| S.Hemishere | 311 | 0.978 | 300 | 0.941 | 263 | 0.825 | 266 | 0.837 | 241 | 0.758 | 243 | 0.762 |
| I. Hemisphere | 309 | 0.972 | 300 | 0.944 | 273 | 0.858 | 249 | 0.781 | 262 | 0.823 | 236 | 0.741 |
| Tempo | 301 | 0.473 | 296 | 0.465 | 262 | 0.412 | 243 | 0.382 | 247 | 0.388 | 230 | 0.361 |
| Superior | 318 | 0.5 | 302 | 0.475 | 267 | 0.419 | 280 | 0.439 | 244 | 0.383 | 260 | 0.409 |
| Nasal | 312 | 0.491 | 301 | 0.72 | 265 | 0.416 | 246 | 0.387 | 242 | 0.380 | 221 | 0.348 |
| Inferior | 310 | 0.487 | 301 | 0.473 | 278 | 0.436 | 261 | 0.410 | 274 | 0.430 | 245 | 0.385 |
|  |  |  |  |  |  |  |  |  |  |  |  |  |
| Perifovea | 289 | 3.632 | 278 | 3.493 | 247 | 3.105 | 255 | 3.204 | 238 | 2.993 | 225 | 2.826 |
| S.Hemishere | 295 | 1.853 | 277 | 1.742 | 248 | 1.557 | 258 | 1.621 | 226 | 1.421 | 232 | 1.46 |
| I. Hemisphere | 283 | 1.779 | 279 | 1.751 | 246 | 1.547 | 252 | 1.583 | 250 | 1.572 | 217 | 1.365 |
| Tempo | 281 | 0.882 | 273 | 0.859 | 250 | 0.784 | 242 | 0.762 | 246 | 0.772 | 217 | 0.682 |
| Superior | 299 | 0.939 | 277 | 0.870 | 244 | 0.768 | 258 | 0.811 | 240 | 0.755 | 234 | 0.734 |
| Nasal | 300 | 0.943 | 290 | 0.911 | 249 | 0.783 | 255 | 0.801 | 225 | 0.706 | 231 | 0.727 |
| Inferior | 276 | 0.868 | 271 | 0.853 | 245 | 0.771 | 264 | 0.831 | 242 | 0.760 | 217 | 0.683 |
|  |  |  |  |  |  |  |  |  |  |  |  |  |

Abbreviations: S. Hemisphere: superior Hemisphere; I. Hemisphere: inferior hemisphere; OD: right eye; OS: left eye; Vol: volume; Thick: thickness.

**Supplementary Table S3. Identified genetic variants of USH genes in the proband (VI-2) after filtering out common variants, variants in introns, and synonymous mutations.**

| **Gene Name** | **c.** | **p.** | **Splicing** | **Type** |
| --- | --- | --- | --- | --- |
| USH2A | c.10312G>A | p.Ala3438Thr | exonic | homo |
| USH2A | c.11389+15->A | - | splicing | het |
| USH2A | c.6379_6380delinsC | p.Gly2127Profs*25 | exonic | homo |
| USH2A | c.11389+14->A | - | splicing | het |
| WHRN | c.1352G>A | p.Gly451Asp | exonic | het |

Abbreviations: homo: homozygous; het: heterozygous.

**Supplementary Table S4. Identified genetic variants of USH genes in family member VI-3 after filtering out common variants, variants in introns, and synonymous mutations.**

| **Gene Name** | **c.** | **p.** | **Splicing** | **Type** |
| --- | --- | --- | --- | --- |
| WHRN | c.716C>T | p.Pro239Leu | exonic | het |
| USH2A | c.10312G>A | p.Ala3438Thr | exonic | het |
| USH2A | c.9958G>T | p.Gly3320Cys | exonic | het |
| USH2A | c.8284C>G | p.Pro2762Ala | exonic | het |
| USH2A | c.6379_6380delinsC | p.Gly2127Profs*25 | exonic | het |

Abbreviation: het: heterozygous.
